# Validation of computational models simulating injury-related kinematics with muscle activation – obtaining data under general anaesthesia

**DOI:** 10.1007/s00414-025-03577-0

**Published:** 2025-08-12

**Authors:** Lea Siebler, Sarah Thaler, Julia Muehlbauer, Steffen Peldschus, Philipp Groene, Sylvia Schick, Simon T. Schaefer

**Affiliations:** 1 Biomechanics and Accident Analysis, University of Munich LMU, Munich, Germany; 2 Clinic for Anaesthesiology, University of Munich LMU, Munich, Germany; 3 Intensive Care Medicine, Emergency Medicine and Pain Therapy, University Clinic for Anesthesiology, University Medicine Oldenburg, Carl von Ossietzky University and Clinic Oldenburg, Oldenburg, Germany

**Keywords:** Validation experiments, Passive kinematics, Relaxation, Knee flexion test, General anaesthesia

## Abstract

Reconstructing injury-related movements and reactions in forensic biomechanics (e.g. abusive head trauma, whiplash, or falls) requires biofidelic representations of the human body. Muscle tone and activity can be integrated into these computational human body models. However, obtaining high-quality data on passive joint behaviour, which is crucial for ensuring accurate simulation results, remains challenging. A challenge in volunteer experiments is the high kinematic variability affecting passive responses. This study investigates differences in gravity-induced knee flexion between anaesthetised and awake subjects, aiming to clarify how muscle tone influences passive behaviour. Knee flexion kinematics and vastus lateralis activity were measured in eleven patients scheduled for surgery. Three tests were performed while patients were awake, two following propofol sedation, and three after administering a muscle relaxant. In awake trials, median duration to reach 47° knee flexion was 404 ms, compared to 355 ms for anaesthetised and 349 ms for anaesthetised + relaxed. Significant differences were found between anaesthetised + relaxed (p = < 0.001) and between anaesthetised + relaxed versus anaesthetised (*p* = 0.004). Only 15% of awake trials showed no measurable muscular activity. The results indicate significant muscle tension that cannot be detected by EMG and cannot be suppressed in awake patients. This study explores the differences in gravity-induced knee flexion between anaesthetised and awake subjects, focusing on how muscle tone impacts passive responses amidst the challenges of high kinematic variability. This study offers reference data for future validation and shows how involuntary activity in non-anaesthetised subjects can enhance computational models for addressing scenarios like abusive head trauma or falls.

**Supplementary Information:**

The online version contains supplementary material available at 10.1007/s00414-025-03577-0.

## Introduction

The reconstruction of injury-related movements and reactions, as typically addressed in forensic biomechanics, requires representations of the human body that are able to incorporate the effects of muscular activity [1]. The body’s responses, for instance, are crucial in the context of falls, whiplash-associated disorders or abusive head trauma/shaken baby syndrome, where the kinematics produced by active as well as passive movements in the analyzed event determine the potential injury outcome. The question on potential contribution of muscular action is even more difficult to analyze in situations of unconsciousness, heavily alcoholized or drugged person, or in general in case of weak musculature. Muscle tone and activity, distinct from passive biomechanical behaviour, can be included in computational models of the human body. In the future, this may offer the opportunity of analyzing or demonstrating the effect of potentially (in)active muscles or acting forces. As passive muscles contribute to the resulting motions of the human body to some extent, experimental data is needed for the development of simulation models. However, acquiring high-quality reference data for purely passive behaviour of human joints constitutes a particular challenge, while it is essential for the level of trust one can develop in simulation results.

Musculoskeletal models of the human body have numerous applications in ergonomics, sports biomechanics, and vehicle safety research [2–4]. Computational models of the human body and their underlying validation data have been presented in the context of forensic practice [5–8]. To gain insight into the role of a model’s muscle in kinematic behaviour under load, a comparison with human responses at various muscle activity levels is necessary. Although intentional muscle activity can be controlled voluntarily, intentional muscle relaxation is more difficult to achieve. Biomechanical studies of muscle mechanics conducted with subjects awake and/or under general anaesthesia (for clinical studies based on data from healthy control groups) [9–13], necessitate questioning the exclusive dependence on awake volunteers for robust musculoskeletal model validation data regarding joint stiffness. These findings from the literature suggest that passive kinematics, particularly in the context of musculoskeletal model validation, generally cannot be acquired from self-reported relaxed, awake volunteers. Cadaver tests are a method for investigating behavior during movement without the interference of muscle activity. However, since correlating passive behavior to voluntary limb movement is essential, it is important to compare the data within an individual against scenarios where muscle control is either voluntary or reflexive.

The present study conducted new experiments to collect proven passive kinematics for passive model validation. The experiments were conducted with patients undergoing scheduled surgery that required general anaesthesia.

A standardised gravity-induced leg flexion test, originally developed by Wartenberg [14], is well-established as a clinical procedure for assessing muscle tone. To conduct this test, the unloaded lower leg of a sitting or supine person with a fully extended knee can drop freely. Based on this procedure, van der Meché and van Gijn narrowed down a dataset of 72 supposedly completely relaxed lower leg drop trials to six truly passive trials, verified through vastus lateralis surface muscle activity [9]. In the manner of van der Meché and van Gijn’s experimental setup, Muehlbauer et al. [15] conducted a novel series of gravity-induced knee flexion tests designated specifically for model validation. Nine healthy male volunteers, matched in anthropometry and age, performed sixteen lower leg drop tests, each aimed at not actively resisting or assisting the passive motion of the knee joint. The subjects generally displayed high intra- and interindividual muscle activity and kinematic variations. In line with observations by Fee and Miller [12, 16], muscle relaxation was difficult for many healthy volunteers to achieve while awake. Nevertheless, the study replicated van der Mechè and van Gijn’s findings by successfully identifying nine out of 135 trials in which volunteers could relax to a level where only extremely low muscle activity was discernible by standard EMG.

In theory, the individual fall times of a physical pendulum can be calculated and compared to those of relaxed volunteers to estimate the remaining combination of muscle tone and joint stiffness. However, this calculation needs information on the centres of gravity of the lower leg and foot complex, which must be determined by analysing the tissue densities. This is difficult to measure precisely in living humans.

As indicated by McKay et al. [11], we hypothesise that the kinematics of anesthetised subjects under the influence of muscle relaxants differ significantly from those of awake, self-reported relaxed subjects. This is based on the assumption that awake volunteers cannot sufficiently relax their muscles. We assume an absolute minimum level of muscle activity (baseline passive kinematics) suitable for musculoskeletal modelling. Thus, it is assumed that under anaesthesia, with fully relaxed muscles, the passive stiffness of the joint response and soft tissue can be observed in a standardised and reproducible experimental setup. In addition to testing in both the awake and anaesthetised + relaxed states, trials in the anaesthetised state prior to muscle relaxant administration were included. These experiments aimed to gain insight into whether there is a difference in kinematics between anaesthetised and anaesthetised + relaxed patients.

## Methods

The study employs a prospective, observational design. All testing procedures received approval from the Ethics Committee of the Medical Faculty of the University of Munich LMU, Germany (approval number: 20–203). To qualify for participation, patients needed to be between 18 and 70 years of age and planning to undergo surgery that required general anaesthesia. Patients with a known history of neuro-muscular-skeletal diseases or symptoms were excluded from enrolment. The selected study population consisted of six female and five male patients scheduled for elective abdominal surgery. All of them gave informed consent for their participation. None of the patients received any pre-medication, such as tranquillizers, prior to the test series. Table 1 provides key values of the study cohort’s anthropometric distribution. Age was not normally distributed in either sex and body weight was not normally distributed in males; refer to Table 1. Individual values can be found in the Supplementary Information Table 2.

**Table 1:** Anthropometric distribution of eleven patients (six female, five male) age, body height (cm), body weight (kg) and body mass index (BMI) including minimum (min), 25th, 50th and 75th percentile and maximum (max)

	Sex	Min	25th	Median	75th	Max	Shapiro-Wilk, *p*-value
Age (years)	female	31	37.75	57.5	58	58	0.010
	male	26	28.5	59	62	63	0.063
Body height (cm)	female	162	167.25	175.5	180.25	181	0.522
	male	170	171.5	175	184.5	187	0.661
Body weight (kg)	female	52	56.5	81	90.5	92	0.218
	male	60	67.5	75	78.5	80	0.063
BMI (kg/m²)	female	16.6	19.38	25.34	31.08	33.2	0.868
	male	19.6	20.52	23.25	26.37	27.7	0.981

Patients were required to lie supine on the operating table to ensure continuous medical access. For testing, the left leg was positioned comfortably on the operating table; the right heel rested in a repeatable and comfortable position on the foot support, while the lower leg remained unsupported. The height of the foot support was adjustable to initially load the thigh, minimising soft tissue motion due to gravity after the release of the support, thereby isolating the motion of the lower leg. A consistent initial knee angle, measured between the lateral malleolus, the lateral knee joint cavity, and the greater trochanter was established for each trial to eliminate the potential risk of knee joint injury and to facilitate reproducible testing conditions. A non-removable heating blanket was required for the surgery of six of the eleven patients (P01, P02, P03, P04, P06, P09). Safety measures, including the securement of the foot support after the first swing and the cushioning of contact areas were explained and demonstrated to the patients to boost their confidence and encourage relaxation. Each trial commenced with the release of the foot support using a manual switch, triggering the motion recording and the simultaneous start of the EMG measurement. Before initiating a trial, a three-second countdown (spoken aloud) was given to raise awareness, ensuring that the staff in the operating room were alerted to the start of the test.

The test matrix included eight gravity-induced knee flexion tests for each patient at three different stages:

1. Three trials while awake and without premedication as an awake control reference (hereafter referred to as Awake Control (C)).
2. Two trials after the induction of anaesthesia and before the administration of muscle relaxant (hereafter referred to as Anaesthetised (A)).
3. Three trials under anaesthesia after the administration of muscle relaxant (hereafter referred to as Anaesthetised + Relaxed (AR)).

Before and during each test run in the awake control condition (C), patients were instructed to relax completely and not to intervene or actively support passive knee flexion.

Patients were administered general anaesthesia using propofol and sufentanil, based on clinical needs. Propofol was given at a dosage of approximately 2-2.5 mg/kg body weight, while sufentanil was dosed at about 0.2–0.4 µg/kg. Only two lower leg drop trials could be conducted during this interval phase (A) to not prolong the time of anaesthesia induction. Subsequently, a muscle relaxant (rocuronium) was administered, dosed according to the Train of Four count. Following the attainment of a Train of Four of zero, intubation and mechanical ventilation ensued. The mean dosage of the initial propofol bolus was 2.58 ± 0.59 mg/kg (1.88; 3.73). The diffusion rate via a syringe pump was 5.51 ± 1.22 mg/kg/h (4.00; 7.69). Detailed information on propofol dosages is available in the Supplementary Information Table 3. The Train of Four was assessed in the AR condition to confirm complete muscle relaxation. This method is commonly employed to monitor muscle relaxation when muscle relaxants are used in anaesthesia [17]. Therefore, patients underwent knee flexion tests with confirmed complete muscle relaxation during the AR trials. The entire testing procedure did not add significant time to the patients’ surgery beyond what was medically necessary.

Surface EMG signals were recorded for the vastus lateralis muscle (VL) at 2000 Hz (Myon 320, Myon AG, Schwarzenberg, Switzerland; NI USB-6210 A/D converter, National Instruments, Austin, TX, USA) with an input range of 1.25 V using proEMG software (version 2.1, ProPhysics AG, Switzerland). Before attaching the surface electrode (Ambu BlueSensor N, Ambu A/S, Ballerup, Denmark) in accordance with SENIAM recommendations [18], the skin was shaved and cleaned with abrasive paste to maximise electrical conductivity. EMG data were band-pass zero-phase filtered using the Butterworth method with cut-off frequencies of 20 and 500 Hz, rectified, and root mean square smoothed using a 25 ms window (MATLAB, version R2020b, Mathworks Inc.). To assess muscle onset and offset times, statistical (root mean square) significant change points of signal characteristics were utilised [19, 20]. As measurements of maximum voluntary muscle contraction (MVC) could not be incorporated into established medical procedures, human resting muscle tone (HRMT) was recorded as a normalisation reference prior to the test series in the operating theatre. The processed EMG signal magnitude is expressed as a multiple of the individual HRMT from the resting awake EMG signal. Based on the measurements gathered before this test series, the HRMT was determined as the lowest average value within a floating window of 150 ms following processing. This was assumed to represent the lowest activity achievable by the patient.

**Fig. 1:**
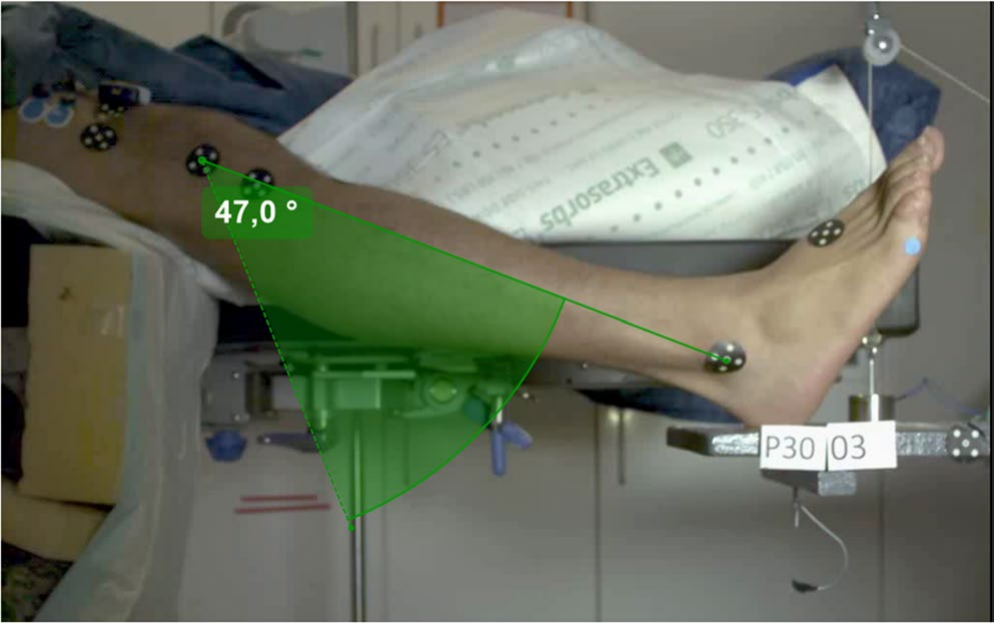
Illustration of lower leg fall angle calculation shown for 47 ° knee flexion with the centre of rotation at the lateral knee joint marker and the initial position of the malleolus marker set as 0 (solid line) as reference for differences of lower leg kinematics between awake, anaesthetised, and anaesthetised and muscle relaxed state, respectively

Sagittal lower leg displacement was captured at 2000 Hz using a high-speed camera (Photron FASTCAM SA-Z, 36-bit colour version). Skin markers were placed on the right os metatarsal V, the arch of the foot, the lateral malleolus, the caput fibulae, the tibial tuberosity, the patella, the lateral knee joint, and two markers in a vertical line on the lower third of the thigh. The video quality was enhanced using Photron software (PFV, version 3630). The trajectories of the markers were tracked with the freeware Kinovea (version 0.8.15, Joan Charmant & Contrib.) and manually corrected as necessary. The knee angle was assessed relative to the individual starting position for each trial, utilising the markers on the lateral knee joint and the lateral malleolus, resulting in knee kinematics beginning at coordinates 0/0 at time zero. The most comprehensive data set was obtained for a knee angle of up to 47°, which is why this angle was employed for further evaluation. In Fig. 1, the approximation of the fall time for the 47° knee angle is displayed. Possible leg and foot positioning changes were visually inspected and documented throughout the various states to note any irregularities. A more precise measurement was unattainable due to limitations of the setup, ensuring efficient realisation of the test during the induction of anaesthesia.

### Statistical analysis

To analyse intraindividual differences resulting from states of consciousness, the median fall times until knee flexion of 47° from the three test repetitions (only two in the anaesthetised condition) were recorded for each individual and each state. A Friedman test for related samples was conducted. The significance level was established at 5% and adjusted for the pairwise comparisons (1.7%) in accordance to Bonferroni. Furthermore, this test was performed solely for patients and trials with relaxed VL muscle in the awake state.

To investigate potential differences in fall time values among patients in the AR state (using a single median value), we examined the effects of various factors: body height, body weight, BMI, sex, the use of a heating blanket, lower leg length^1^, lower leg mass^2^, the square root of lower leg length, and lower leg circumference. The Wilcoxon Mann-Whitney U-test was utilized for independent samples (specifically sex and heating blanket), while the Spearman Correlation coefficient was calculated for the continuous variables. The bivariate correlations among the variables are examined to select independent input variables for the statistical modelling of the fall times. A linear regression model with backward elimination is calculated by selecting independent variables associated with median fall times in the AR states.

## Results

A total of 86 trials were conducted, three of them lacking a video signal due to technical issues (P08 A1, P03 AR2 and P06 AR2). The EMG data is complete. For one patient (P10), only one test was carried out for state A to ensure an uninterrupted medical workflow. The initial leg positions demonstrated low variance with a knee angle of 158 ± 3° calculated from the video footage and averaged across all trials.

**Fig. 2:**
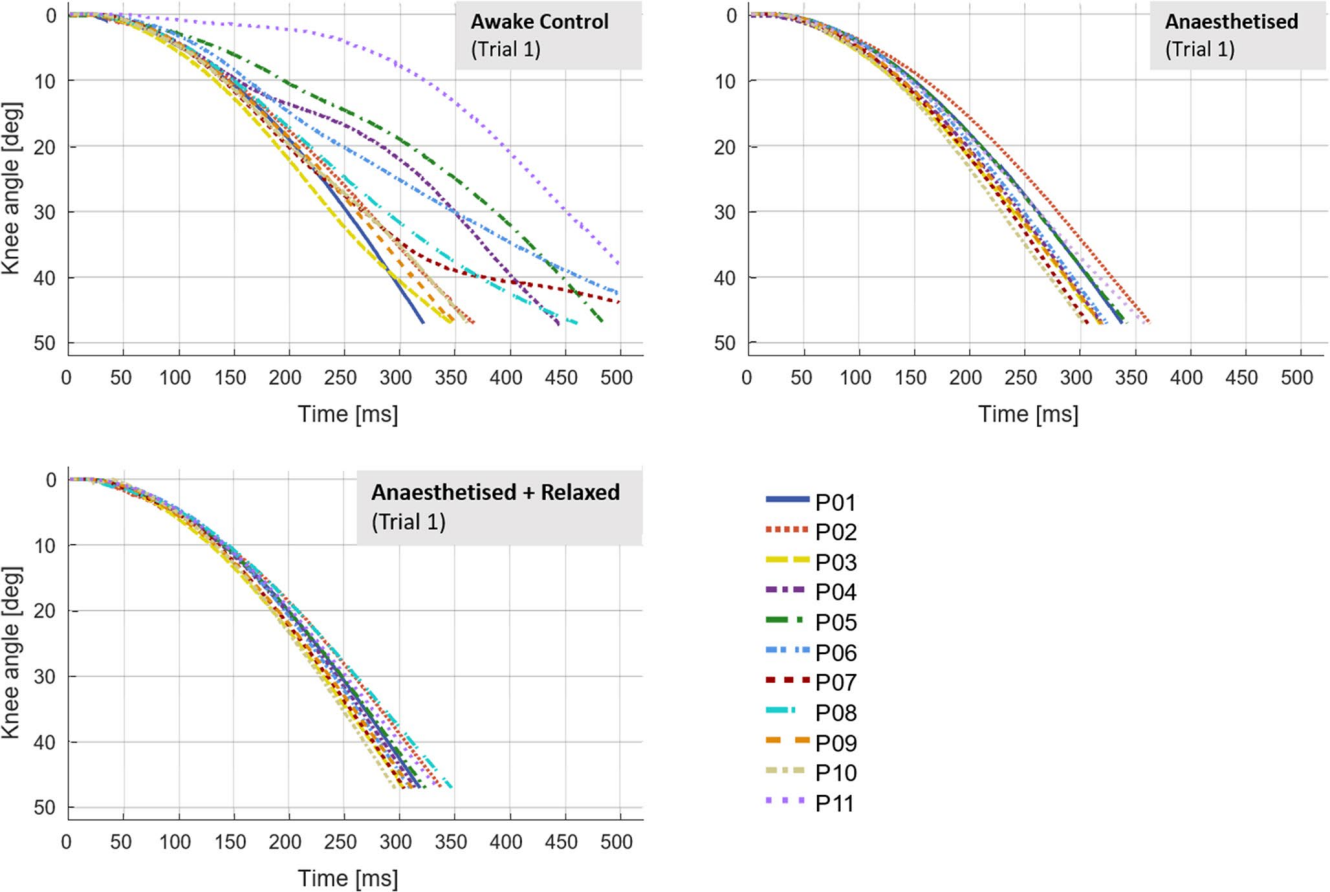
The individual sagittal knee angle time course (°) of the patients in the first trial of each condition (awake control state, the anaesthetised state, and the anaesthetised + relaxed state). The knee angle was determined until reaching 47° relative to the knee angle at the start time (0°)

The kinematic data shows high interindividual variability in C (see Fig. 2). This variability diminishes following the induction of anaesthesia and decreases further in state AR. Figure 2 illustrates the individual knee angle time courses from the first trial under each condition (C, A, and AR). Additional time course plots of knee angle for the other trials are available in the Supplementary Information (Fig. 8).

**Fig. 3:**
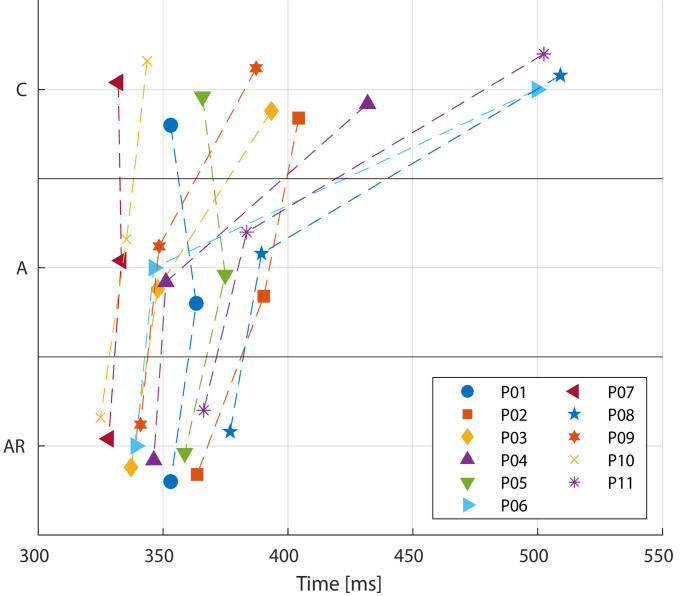
Median lower leg fall times (ms) until 47° knee angle for all patients during the three states: awake control (C), anaesthetised (A) and anaesthetised + relaxed (AR)

Compared to the trails in state C, the median fall time of the lower leg at 47° decreased for A and AR. In state C, the median fall time was 404 ms, ranging from 323 ms to 672 ms. In the states A and AR, the median fall time decreased to 355 ms (327 ms to 402 ms) and 349 ms (322 ms to 385 ms), respectively. The Friedman test for related samples indicated significant differences in the fall times at a 47° knee angle across the three states (*p* = 0.000). Pairwise comparisons revealed significant differences between states C and AR (*p* = 0.000) and between states A and AR (*p* = 0.004); however, there was no significant difference between states C and A (*p* = 0.337). The median fall time values for each patient across the three states are illustrated in Fig. 3.

The fall times of all patients in both states C and A did not exhibit a normal distribution (Shapiro-Wilk *p* < 0.200). The median fall times in the AR state were normally distributed; however, they still differ between individuals. These individual median fall times in AR showed a moderate to strong association with sex, age, body height, BMI, lower leg circumference, and lower leg length (Spearman correlation coefficients > 0.5 and p-value less than 0.2), but not with the heating blanket, lower leg mass, or body weight. Intercorrelations existed, particularly between sex and lower leg circumference, as well as between body height and lower leg length. Linear regression with backward modelling resulted in the remaining variables age, body height, and lower leg circumference (coefficients: age: −0.29, body height: 1.399, lower leg circumference: −1.634, constant: 177.645, and R²=0.940).

**Fig. 4:**
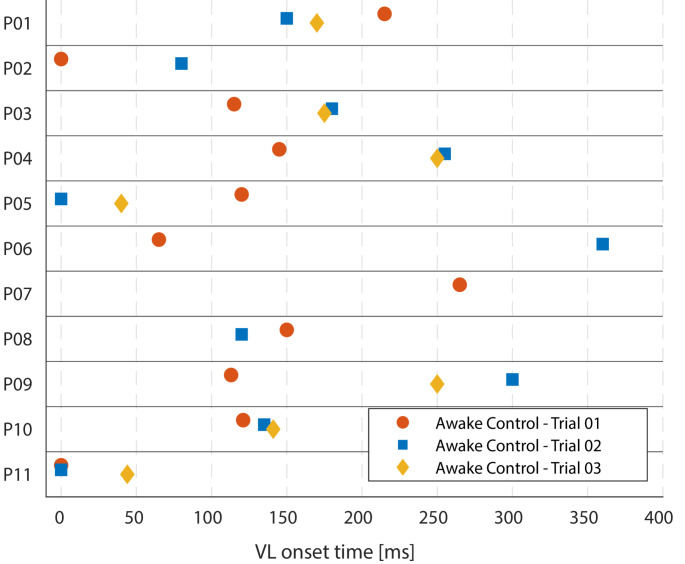
Individual Vastus lateralis (VL) activity onsets (ms) across the first three trials (awake control state, (C1-C3)) for patients P01-P11 (*n* = 28; in five trials no onset was detected)

In 28 out of 33 awake trials (85%), muscle activity was evident in the Vastus Lateralis (VL) EMG signal, characterised by its onset, peak, and offset. The onset time of the VL during C ranged from 0 ms to 360 ms. In three trials (P05 in C1, P11 in C1 & 2), the EMG activity commenced simultaneously with leg motion. The corresponding times to onset for the VL are shown in Fig. 4. The peak magnitude of VL activation during the awake control trials consistently exceeded the HRMT level by at least two times, with a maximum of 32 times the HRMT. No clear trend could be observed regarding the sequence of muscle activity magnitudes across the three awake control trials.

**Fig. 5:**
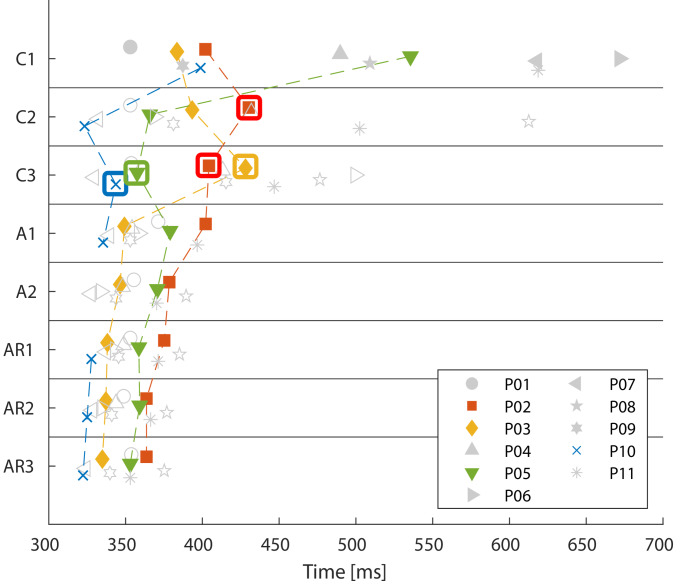
Lower leg fall times (ms) until 47° knee angle for all patients for every trial in the three states: awake control (C), anaesthetised (A) and anaesthetised + relaxed (AR) for patients P01-P11. Trials in the awake control condition (C) that are classified as vastus lateralis (VL) passive are marked with a box. Patients without a trial classified as VL passive in the awake control condition are displayed in light grey

In the first trial of state C there was no trial without VL activity; however, there was one in the second trial (P02) and four in the third trial (P02, P03, P05, P10). The corresponding fall times are classified as VL passive and are indicated with a box in Fig. 5. The passive fall time of P05 in C was 358 ms, making it close to the fall times in the AR state (AR1 359 ms, AR2 359 ms, AR3 353 ms), but it differed from the fall times in A (A1 379 ms and A2 371 ms). For all other patients, the fall times classified as VL passive deviated from the median fall times in the AR state. In the passive C trials of P02, the fall time was 41 ms higher than the individual median fall time under AR; the same applies to P03 and P10, which were 91 ms and 14 ms higher, respectively. Only P05 demonstrated a longer fall time duration during A compared to the awake control condition. No clear trend could be observed regarding the sequence of muscle activity magnitudes across the three trials of the C condition. P02 exhibited comparable fall times in the first and third awake trials as well as in the first trial of A. However, the fall times in trials C2 and C3 without VL activity differed.

**Fig. 6:**
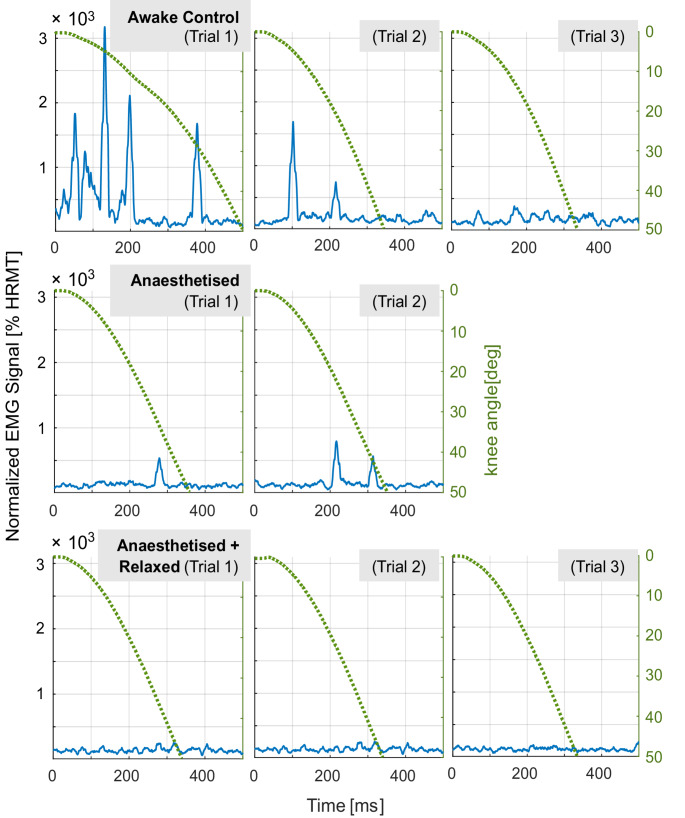
Filtered and normalized EMG activity of the vastus lateralis (VL) (blue) and the sagittal knee angle time course (°) (green dotted) for the three trials in the awake control condition for patient 05. The sagittal knee angle is shown until 47° were exceeded while the corresponding EMG signal is shown for 500 ms. Trial 3 was classified as VL passive

The four patients (P02, P03, P05, P10) with at least one VL passive trial in state C showed median fall time durations until 47° knee flexion in those passive trials of 387.4 ms (range 343.6 ms; 428.1 ms) in C, 361.4 ms (range 335.4 ms; 390.3 ms) in A, and 347.6 ms (range 325.0 ms; 363.6 ms) in AR. There were no significant differences when comparing the VL passive C trials to the corresponding state A (*p* = 0.724) and AR (*p* = 0.077), nor between the two anaesthetised states (*p* = 0.157). In state A, where no muscle relaxant had yet been administered, low EMG activity was evident during the lower leg drop in one case (P04) in trial A2. The EMG was silent after the Train of Four showed no response, indicated in the AR condition. Figure 6 illustrates the VL’s knee angle time course and corresponding EMG data during all trials (Trials C1-3, A1 + 2, AR1-3) of P05. The trail C3 was classified as VL passive for this patient.

**Fig. 7:**
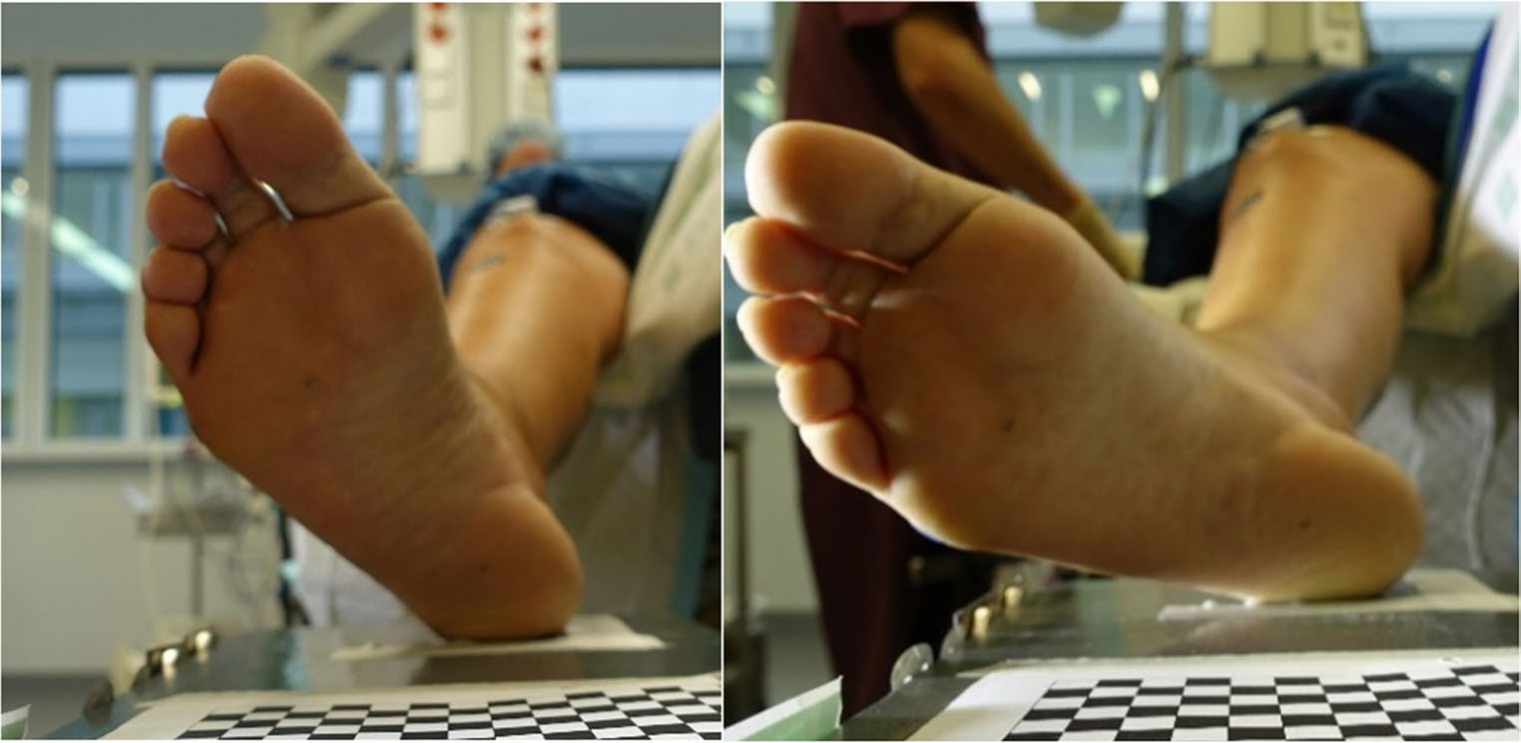
Exemplary initial heel position of the same volunteer awake state C (left) and after muscle relaxant administration (state AR) (right)

Furthermore, external rotation of the leg was noted. There was a visible increase in external rotation from the trials during C to those during AR (Fig. 7). However, this was not specifically measured; it was merely an observation made by the investigators. This observation of increased external rotation could be seen in nearly all patients.

## Discussion

The study provides valuable results for the further development of computational tools in the field of forensic biomechanics. The findings on activation levels in volunteer experiments compared to purely passive experiments allow to design more precise experiments in the future. In addition, the obtained data may serve as target data for computational tools in order to prove their validity. This paves the way to the development of simulations of forensically relevant scenarios under low accelerations and low-energy loading with passive or partly active musculature such as abusive head trauma.

In general, lower-leg fall times decreased with increasing levels of anaesthesia. Additionally, lower-leg fall times exhibited considerable inter- and intraindividual variability in the awake state, decreasing with deeper general anaesthesia. Prolonged fall times in state C were related with activity of the VL. Few subjects demonstrated passive behaviour in state C, as indicated by the muscle activity of the VL. Statistics revealed significant differences between individual states, notably between the state C and AR, as well as between state A and AR. The lack of significant differences between the state C and A can be attributed to some individuals showing longer fall times in A compared to C, which may be explained by the supportive behaviour of the knee flexors, for which no EMG data were available in this study.

However, the results revealed a persistent interindividual variability in the fall times during AR. In this state, the median fall time of 349 ms varied by 63 ms between the minimum and maximum durations recorded for individual patients. In comparison, there was only a median difference of 6.1 ms (range 3.3 ms to 18.2 ms) in the trials of respective patients. The regression model indicates that around 90% of this interindividual variance could be explained by factors such as age, body height, and lower leg circumference, the latter being closely associated with sex. Moreover, differences between individuals might be due to factors like the rotational dynamics of the hip joint, characteristics of passive tissues or joint friction, and the varying distribution of mass in the lower leg and foot, all contributing to the individual physical pendulum’s length Individual anthropometrics and tissue characteristics must also be accounted to predict fall times accurately; however, these measurements can be challenging to obtain in living subjects. In this context, an extension of the study involving cadavers may provide a more precise approach to addressing this uncertainty.

Nonetheless, overall kinematic variability was greater in the awake state than under general anaesthesia, both before and after administering the muscle relaxant. This is supported by the presence of EMG activity in the VL during most C trials. At a knee angle of 47°, the median fall times were shorter under general anaesthesia. Based on this observation, it can be assumed that the differences between the awake, unmedicated state and the AR state are due to anaesthesia-induced unconsciousness and muscle relaxation. A comparison of the two states under general anaesthesia did not reveal a statistically significant difference in leg fall time. The trajectories were not compared statistically; however, through visual inspection (see Fig. 6), a delayed muscle activity after 200 ms can be inferred for P05 in state A, as suggested by some minor EMG activity and the comparison of the trajectories in states A and AR (more S-shaped in A, parabolic in AR). To the authors’ best knowledge, only one study has assessed muscle activity in an anaesthetised, non-muscle-relaxed condition (AR) in patients [10]. This study found that the resting muscle showed measurable vibrations and decreased muscle sound under anaesthesia and muscle relaxants. However, the supine M. biceps brachii was used for evaluation, and no kinematic analyses were conducted that could be compared with the present study.

Contrary to McKay et al.’s [11] impression of the short-range flexion tests, but in line with observations by Fee [12, 16] and Muehlbauer et al. [15], muscle relaxation was difficult to achieve for most volunteers in most trials intentionally. Under anaesthesia without muscle relaxants, isolated activities were observed in one trial. During this phase, no measurement of anaesthetic depth was assessed, as anaesthesia was induced clinically based on the anaesthesiologist’s evaluation. Anaesthetic depth could range from deep to light, as some patients received additional injections during this phase. Knee angle time histories did not indicate any trends towards an association between the number of anaesthetic doses and knee kinematics, potentially contradicting a comparison of between-subjects data. Real-life examples of an anaesthetised but not relaxed state of consciousness include unconsciousness or a heavily alcoholised or drugged person. As anticipated, no EMG activity was observed in the AR condition.

In a study published by McKay et al. [11], leg drop tests were carried out with ten patients from a drop height of 16 cm onto a cushioned operating table. This included, among other measurements, EMG measurement of the M. rectus femoris and M. biceps femoris in both the awake state and under general anaesthesia, including the use of muscle relaxants. The authors discovered that paralysis resulted in an increase in acceleration. Because the drop height of the lower leg was constrained to 16 cm in order to assess muscle tone without voluntary activity, this relatively small range of motion limits the applicability of the results regarding musculoskeletal model validation. The results of the current study support the findings of McKay et al. [11] while providing valuable information about VL activity and leg kinematics across a wider range of motion. This offers extended experimental data, including larger motion trajectories. The data from the present experimental study meets the requirements for validation data for musculoskeletal models of the human body, focusing on the differences between model responses with and without muscular activity.

At 47° of knee flexion, the fall times during AR displayed a narrower spread compared to A. Furthermore, the first trial of AR did not visibly differ from the second trial of A. The duration of anaesthesia affected the application of muscle relaxants. Therefore, the additional value of muscle relaxants for passive flexion kinematics is not apparent and may require further investigation. A potential behavioural difference between spinal and non-spinal anaesthesia, as observed by Krabak et al. [13], was not the subject of this study.

In the healthy control groups, data from Fee [12, 16] indicated that approximately 25% of the subjects were unable to perform the leg drop test in a relaxed state while awake. In the present cohort, only five trials were performed without visible EMG activity of the VL. However, knee flexion was observed in all cases. Compared with the success rate of 6.67% (9/135 trials) (i.e. almost passive trials) reported in the laboratory-based test series involving healthy young male volunteers [15], the rate of nearly passive behaviour in the presented clinical cohort appeared unexpectedly high at 15.15% (5/33 trials) in an awake state. When compared to van der Meché und van Gijn’s [9] healthy leg drop cohort (8.33%, 6/72 trials), the present rate can still be considered as high. Initially, the success rate of this study appeared contradictory, as the authors assume that muscle relaxation is typically challenging to attain in patients in unfamiliar clinical environments shortly before undergoing surgery with anaesthesia. In only five trials, four volunteers were able to relax the VL fully. Unlike the laboratory-based test series, but similar to the study by van der Meché and van Gijn [9], the activation of different muscles, specifically M. biceps femoris as antagonist, could not be ruled out in the clinical study presented here. Therefore, it must be assumed that the estimated number of nearly passive trials in the awake clinical cohort was even lower, possibly aligning with the range observed in the laboratory-based study or even less so, owing to the more tense clinical atmosphere. For instance, the fall time of the VL passive trial for P05 was lower than those observed during A. Consequently, for this trial, it can be inferred that knee flexion was actively supported by the biceps femoris. This appears to be why the Friedman test did not achieve significance. As this muscle was not measured, this conclusion can only be drawn from the fall time and the absence of measurable knee extension movement. The individual P02 exhibited differing behaviour and comparable fall times despite variations in VL activities. For these reasons, extended muscle measurements are recommended for the initial anaesthetised trial (agonists and antagonists). The authors would like to emphasise the importance of assessing the muscle activity of at least the antagonist in validation experiments involving awake volunteers.

Leg positioning before and after anaesthesia induction further suggested that awake subjects typically struggle to achieve a completely relaxed posture. As illustrated in Fig. 7, the external rotation in the hip joint increased noticeably in nearly all subjects after the induction of anaesthesia compared to the awake state. The extent to which this external rotation affected the kinematics of knee flexion was not part of this study and was only observed visually. Nevertheless, this should be investigated separately using three-dimensional motion capture. This rotation in the hip joint and the rotational differences in the knee joint may also contribute, alongside the physical pendulum length, to the remaining interindividual variance in the AR condition.

The study has certain limitations and experimental weaknesses. The subject recruitment primarily came from a clinical cohort. Therefore, participants cannot be assumed to be healthy. However, as care was taken to include only patients whose medical conditions did not raise any suspicion of possible interference with the actual task, the authors consider the patients’ health conditions to be negligible. Given the wide range of body height and weight of both sexes, the study may represent a relatively large portion of the population’s anthropometry. However, due to the high level of effort required for testing in a clinical environment, only eleven test subjects could be examined. Although these subjects represent a broad spectrum of the population, the results cannot be generalized directly.

Testing in a clinical environment presented challenges regarding the experimental boundary conditions. The positioning procedure for the right lower leg proved more difficult with the six patients who lay on a heating blanket. However, no association was found with individual median fall times in the AR state. A potential influence on the magnitude of external rotation of the leg in the tested states cannot be ruled out concerning the use of a heating blanket. Therefore, the next step should involve transferring the clinical measurement conditions to a standardised laboratory environment with volunteers to ensure that valuable, truly passive kinematic data can be utilised for validating biomechanical models.

Assessment of MVC for EMG normalization was not possible on the test day. Given the potentially questionable comparability with the EMG activity on test day, MVC recordings were also not conducted before the test day. While HRMT is uncommon, it has been utilised as a normalisation factor for EMG activity in previous studies [22, 23]. To illustrate the magnitude, McKay et al. [11] reported that resting muscle activity is approximately 1.5% of the MVC EMG level. As HRMT was recorded in the operating theatre, the authors cannot completely exclude the possibility that the measurement was free from electrical noise from the patient’s environment. Nevertheless, the analysis of the frequency spectrum of the raw EMG signal did not reveal any evidence of electrical noise interference, so it is assumed that this is genuine resting muscle activity.

When using data as presented in this study or potentially using computational models based on such data, forensic experts should be aware of the associated uncertainties, especially in cases with low impact energies or low acceleration levels as in whiplash scenarios or in pre-crash occupant kinematics. The long durations of such events, together with uncertainties in muscle contribution, constitute important challenges in the analysis of human motion.

## Conclusions

The present experimental study provides new insights into the knee flexion response under different physiological states, namely in the awake state and between the administration of anaesthetics and muscle relaxants. The data can be considered for future validation of computational models and indicate limitations in obtaining the required details for passive-model validation with human volunteers.

The study highlights a concept of baseline passive joint flexion response that differs from the kinematics observed in awake subjects. Anesthesia results have been found to be comparable between A and AR outcomes. A kinematic response close to pure passive flexion appears achievable under anaesthesia, even prior to the administration of a muscle relaxant. This data indicates that an awake, subjectively relaxed individual is insufficient to fully characterise relaxed kinematics. For further analysis of the behaviour of the healthy human body under gravitational conditions, the potential impact of elevated muscle tone during motion on kinematics ought to be considered. HMRT should be included in data acquisition with awake volunteers to better understand which experimental results might serve as the most accurate proxy for passive reference data. To make the present data applicable for passive musculoskeletal modelling, the results should be analysed in comparison to a standardised laboratory environment. Possible shortcomings that require attention include boundary conditions and the standardisation of initial positioning due to experimental conditions imposed by the clinical setting. Moreover, future three-dimensional measurements of passive gravity-induced knee flexion tests could enhance our understanding of the influence of initial lower leg posture on passive knee kinematics in such assessments.

The results support the development of a better understanding of the reactions of the human body to scenarios at low impact energies or low acceleration levels.

The findings of this study should be considered in the enhancement of computational models to address responses in scenarios such as falls, abusive head trauma, whiplash loading or pre-impact reactions in road traffic.

## Supplementary Information

Below is the link to the electronic supplementary material.

Supplementary Material 1 (DOCX 2.19 M)

## Data Availability

The datasets generated during the current study are available from the corresponding author on reasonable request.

## Abbreviations

A: Anaesthetised trials
AR: Anaesthetised and Relaxed trials
BF: Biceps femoris
C: Awake Control trials
EMG: Electromyography
HRMT: Human resting muscle tone
MVC: Maximal voluntary contraction
VL: Vastus lateralis

## Footnotes

1 the sum of malleolus height and the distance from the malleolus to the knee joint.

2 calculated as 4.65% of body mass for males and 4.84% for females, based on Bernstein 1947 as cited by Zatsiorsky et al. [21]
