# Supplementary material for: Validation of computational models simulating injury-related kinematics with muscle activation – obtaining data under general anaesthesia
Source: Int J Legal Med. 2025 Aug 12;139(6):2775–87. doi: 10.1007/s00414-025-03577-0 (PMC12532759; doi:10.1007/s00414-025-03577-0)
Supplement: Supplementary file 1 — Supplementary Material 1 (DOCX 2.19 M) [file 414_2025_3577_MOESM1_ESM.docx]

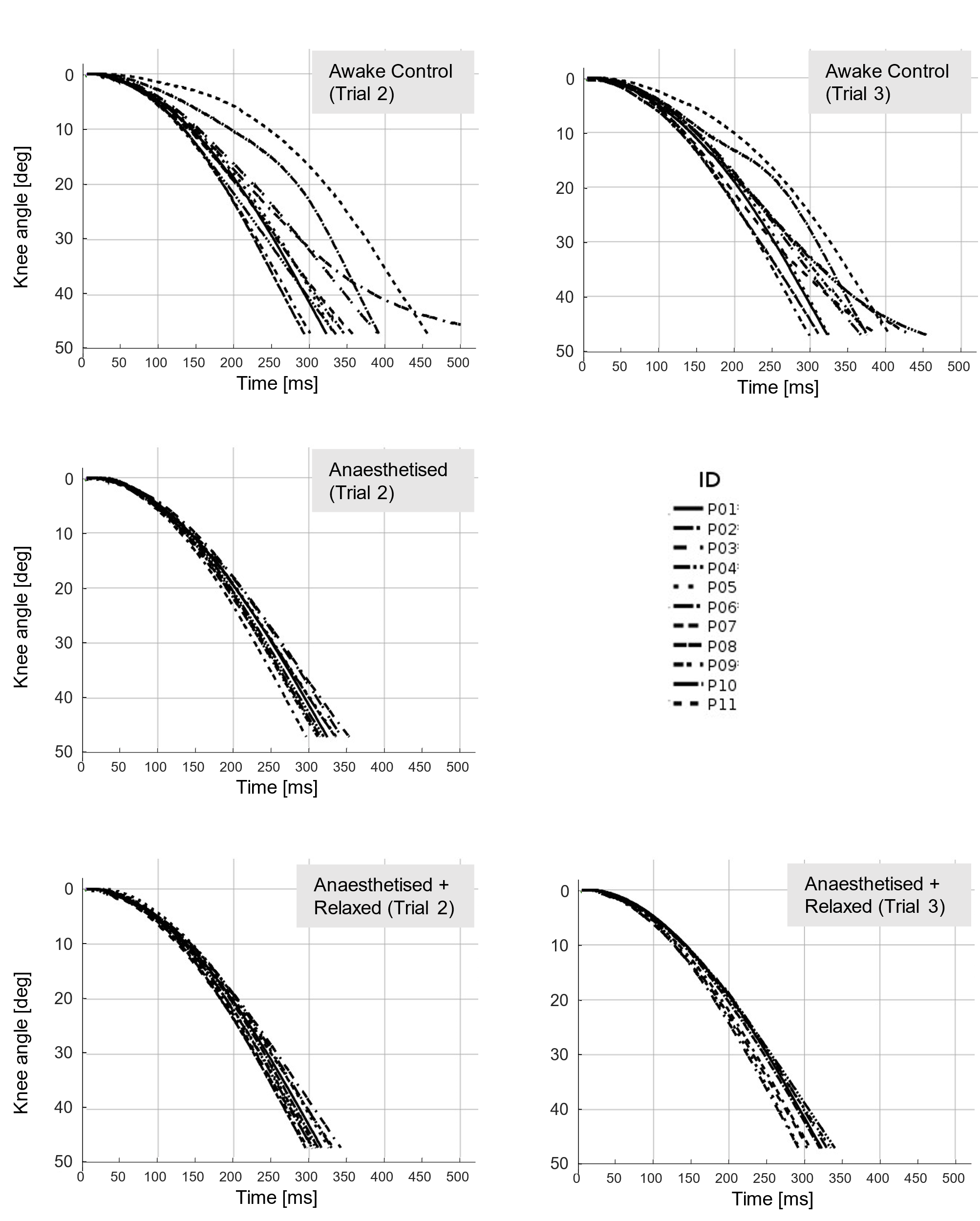


**Fig. 8** The individual sagittal knee angle time course (°) of the patients in trial 02 of each condition (awake control state, the anaesthetized state, and the anaesthetised + relaxed state) and trial 03 in the awake control state, and the anaesthetised+relaxed state. The knee angle was determined until reaching 47° relative to the knee angle at the starting point (0°)

**Table 2** Individual anthropometrics of eleven patients (six female, five male) including age, body height (cm), body weight (kg), lower leg length (cm) and circumference as well as the distance from the Mallellous lateralis to the knee joint (cm). It is also specified for which patients an heating blanket was required.

| ID | Sex | Heating blanket | Age (y) | Body height (cm) | Body weight (kg) | Circumference lower leg (cm) | Distance Malleolus knee joint cavity (cm) | lower leg length (cm) |
| --- | --- | --- | --- | --- | --- | --- | --- | --- |
| P01 | female | yes | 40 | 181 | 75 | 39 | 47 | 54 |
| P02 | male | yes | 31 | 175 | 60 | 30.5 | 44 | 53 |
| P03 | male | yes | 63 | 173 | 75 | 35 | 45 | 52.5 |
| P04 | male | yes | 61 | 170 | 80 | 34 | 42 | 49 |
| P05 | female | no | 31 | 177 | 52 | 31.5 | 44.5 | 49 |
| P06 | female | yes | 58 | 180 | 90 | 43.5 | 40.5 | 53,5 |
| P07 | female | yes | 58 | 162 | 87 | 37 | 40 | 48 |
| P08 | male | no | 59 | 187 | 75 | 29 | 49 | 56 |
| P09 | female | no | 57 | 174 | 92 | 37 | 43.5 | 50 |
| P10 | female | no | 58 | 169 | 58 | 34.5 | 42 | 50.5 |
| P11 | male | no | 26 | 182 | 77 | 37 | 45 | 52.5 |

**Table 3** Amount of propofol administered to patients as bolus and via syringe pump diffusion (mg/kg and mg/kg/hour (h)) (not applicable: N/A)

| ID | Propofol Bolus 1 (mg/kg) | Propofol Bolus 2 (mg/kg) | Propofol Bolus 3 (mg/kg) | Propofol syringe pump 1 (mg/kg/h) | Propofol syringe pump 2 (mg/kg/h) |
| --- | --- | --- | --- | --- | --- |
| P01 | 2.67 | N/A | N/A | 4.00 | N/A |
| P02 | 3.33 | N/A | N/A | 6.67 | N/A |
| P03 | 2.00 | 0.27 | N/A | 5.33 | N/A |
| P04 | 1.88 | N/A | N/A | 5.00 | N/A |
| P05 | 2.31 | N/A | N/A | 7.69 | N/A |
| P06 | 2.22 | 0.33 | 0.56 | 4.44 | N/A |
| P07 | 2.30 | N/A | N/A | 4.60 | 5.57 |
| P08 | 3.73 | N/A | N/A | 6.67 | N/A |
| P09 | 2.17 | 0.33 | N/A | 4.35 | 4.89 |
| P10 | 3.10 | N/A | N/A | 5.17 | N/A |
| P11 | 2.67 | 0.40 | N/A | 6.67 | N/A |
